# Imaging findings of gastric calcifying fibrous tumour

**DOI:** 10.1259/bjrcr.20160064

**Published:** 2016-11-02

**Authors:** Sae Miyashita, Yasuji Ryu, Harumi Takata, Yoshihide Asaumi, Mitsuaki Sakatoku, Takuya Seike, Toshiyuki Okamura, Katsuhisa Inamura, Hiroshi Kawai, Noriko Okuno, Shintaro Terahata

**Affiliations:** ^1^Department of Radiology, Tonami General Hospital, Tonami, Japan; ^2^Department of Surgery, Tonami General Hospital, Tonami, Japan; ^3^Department of Gastroenterology, Tonami General Hospital, Tonami, Japan; ^4^Department of Pathology, Tonami General Hospital, Tonami, Japan

## Abstract

Calcifying fibrous tumours (CFTs) are rare benign lesions that usually affect the soft tissues, the mesentery and the peritoneum. Gastric CFT is particularly rare. Here, we report a CFT found incidentally in a 31-year-old male. The mass was well circumscribed and showed partial calcification on the CT scan, with dark signal intensity seen on T2 weighted MRI. To the best of our knowledge, there is very limited published information concerning imaging findings of CFTs. We discuss the CT scan and MRI findings of this patient, which can be considered typical for gastric CFT, and present a review of the limited literature available.

## Background

Calcifying fibrous tumours (CFTs) are rare benign lesions characterized by hypocellular, hyalinized fibrosclerotic tissue with lymphoplasmacytic infiltrates, lymphoid aggregates, and psammomatous or dystrophic calcifications. CFTs are classically described as soft tissue tumours, but there have been reports of these tumours occurring at other anatomic locations such as the pleura and intra-abdominal organs. Gastric CFT is particularly rare. Here, we report a case of gastric CFT with a review of the literature.

## Case presentation

A 31-year-old male presented to our emergency department with right urinary tract stones. He had no medical history of note and was on no medications. Physical examination of the abdomen was unremarkable. Vital signs and laboratory data were within normal limits.

## Investigations

**Figure 1.**
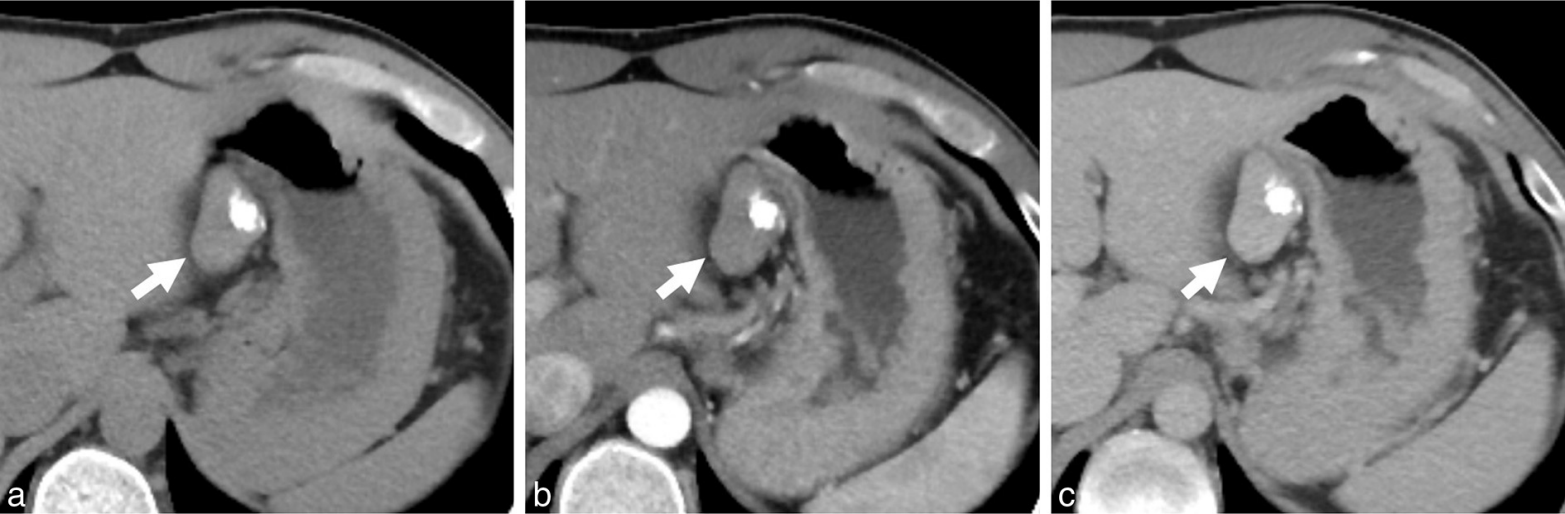
Findings on CT scan. A 36 × 24 mm mass is located in the lesser curvature of the stomach (arrows). The mass is calcified and slowly enhancing. (**a**) Plain image, (**b**) early phase in contrast dynamic study and (**c**) delayed phase.

**Figure 2.**
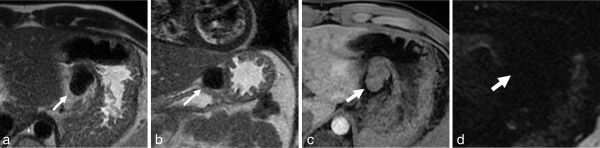
Findings on MRI. Arrows show the mass at the lesser curvature. (a, b) The mass shows a dark intensity on *T*_2_ weighted imaging. (**c**) The mass shows signal intensity almost equal to that of the gastric wall on *T*_1_ weighted imaging, but contains an area of darker intensity. This area was consistent with the calcification seen on the CT scan, so it was considered to indicate calcification. (**d**) The mass does not show restricted diffusion.

**Figure 3.**
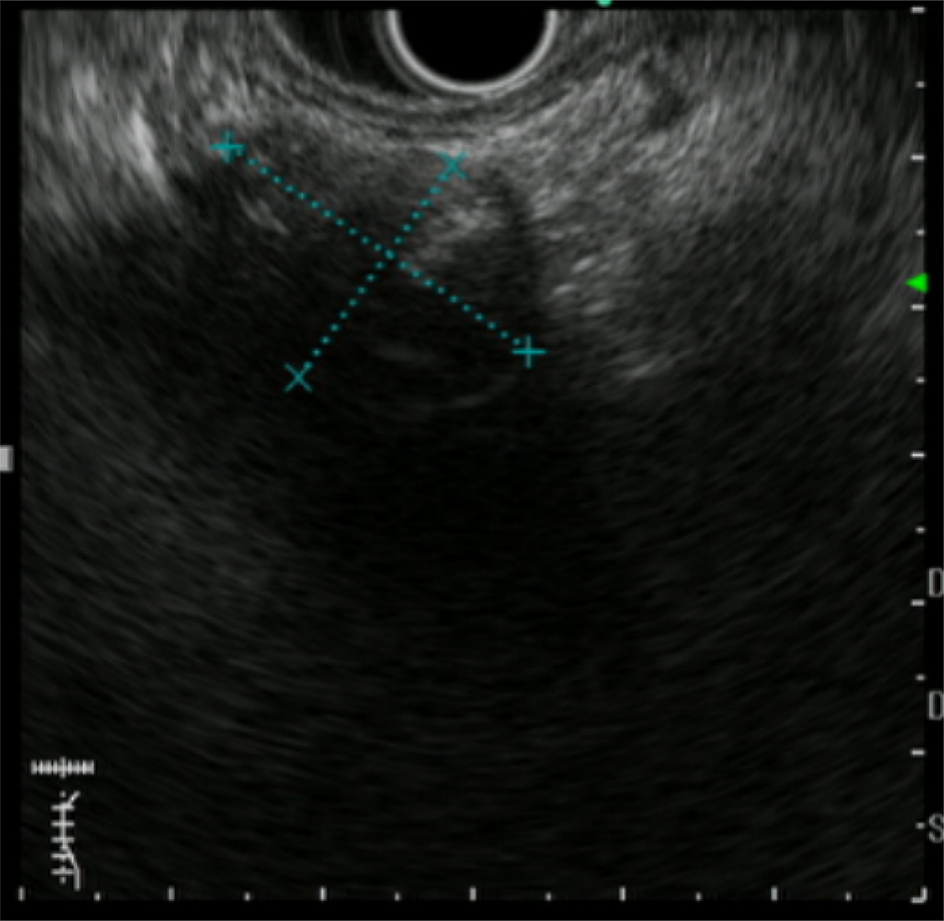
Findings on endoscopic ultrasonography. The mass is a hypoechoic lesion containing a high spot with an acoustic shadow.

A CT scan revealed an incidental, well-circumscribed gastric mass at the lesser curvature. The mass measured 36 × 24 mm and was partially calcified. On dynamic contrast-enhanced CT scan, the mass was slowly enhancing (Figure 1). On MRI, the mass showed dark signal intensity on *T*_2_ weighted images and signal intensity equal to that of the gastric wall on *T*_1_ weighted images. The mass did not show high signal intensity on diffusion weighted imaging (Figure 2). The mass was hypoechoic and cast an acoustic shadow on endoscopic ultrasonography. The hyperechoic component was considered to indicate a calcification (Figure 3). The operator was unable to identify the layer that the mass existed on. The mass was suspected to be a gastrointestinal stromal tumour (GIST) preoperatively on account of this being a more common type of gastric tumour.

## Treatment and outcome

**Figure 4.**
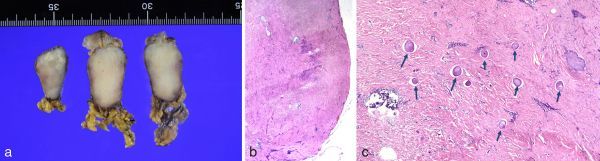
(**a**) Macroscopically, the mass appears white and hard, measuring 3.8 × 2.0 × 2.1 cm. Haematoxylin and eosin staining, with views of low-power field (**b**) and high-power field (**c**). Pathologically, densely hyalinized collagenization with lymphoplasmacytic infiltrates can be seen, and the entire mass is hypocellular. Arrows show many psammoma bodies in the mass.

The patient underwent resection of the mass, which was found macroscopically to be a white, hard, well-circumscribed lesion measuring 3.8 × 2.0 × 2.1 cm. Histological examination showed the mass to be generally hypocellular, with densely hyalinized collagenization, lymphoplasmacytic infiltrates and psammoma bodies, and partially calcified (Figure 4). Immunohistochemistry staining for CD34 showed focal positivity, but was negative for CD117, Dog-1, S100, smooth muscle actin, desmin, anaplastic lymphoma kinase (ALK) and immunoglobulin G4. Therefore, we were able to exclude other gastric tumours such as GIST, schwannoma, leiomyoma or a neuroendocrine tumour, and the patient was diagnosed as having gastric CFT.

## Discussion

CFT is a rare, benign mesenchymal lesion first identified by Rosenthal et al^1^ in 1988 in the limb of a young female and described as “a fibrous tumour with psammoma bodies”. Since 2008, CFT has been classified by the World Health Organization as a benign fibroblastic/myofibroblastic tumour.

CFT shows a predilection for subcutaneous soft tissue of the extremities, and occurs mainly in children and young adults. CFTs have also been reported at other sites, including the neck, pleura, pelvis, liver, mesentery and adrenal glands. However, cases of CFT occurring in the stomach wall are rare, with very few reports in the English literature to date.

Agaimy et al^2^ have published data on seven cases of CFT occurring in the stomach, reporting that the average age at time of discovery was 53 (40–77) years, with a male to female ratio of 1.3 : 1 (four males and three females). The lesions were seen most frequently in the body of the stomach, with an average size of 2.2 (1–3) cm.

Histologically, CFT is characterized by hypocellular, densely hyalinized collagenization with lymphoplasmacytic infiltrates, and forms psammoma bodies.^3^ On immunostaining, this tumour shows positivity for vimentin and factor 13, and often shows focal positivity for CD34. On the other hand, immunostaining for CD117, c-Kit, desmin, actin, S100, smooth muscle actin and anaplastic lymphoma kinase is negative, which distinguishes CFT from GIST, schwannoma, leiomyoma, inflammatory myofibroblastic tumour (IMT) and other gastric tumours.^4,5^

The cause and pathogenesis of CFT are unclear. In the field of pathology, it had been postulated that CFT may be the sclerosing stage of an IMT. Hill et al^6^ compared the histological and immunohistochemical profiles of seven CFTs and IMTs, and identified some differences between these tumours. Other reports have also compared CFTs and IMTs, but none has been able to confirm an association.

CFT is often positive for IgG4, leading some researchers to suggest that CFT might be an IgG4-related disease. However, there have been several reports of CFT being negative for IgG4, as in our case. CFT is no longer considered an IgG4-related disease, but further study is required.^7^

Reports on imaging findings related to CFT are rare, and although we found reports of MRI findings for CFT occurring in the soft tissue, liver and adrenal glands,^8–10^ we found none for gastric CFT. However, we did find some features on CT scan and MRI in our case that were similar to those in previous reports. Partial calcification is seen on MRI in most cases, with a well-circumscribed shape and slow enhancement on dynamic contrast-enhanced studies. The characteristic MRI finding is a mass showing extremely dark signal intensity on *T*_2_ weighted images. These findings are considered to reflect the histological features of CFT, that is, hyalinized collagenization or hypocellularity. In particular, the extremely dark signal intensity seen on *T*_2_ weighted imaging of the entire mass is not seen with other gastric tumours, including GIST, and might be useful for differentiating CFT from other gastric tumours.

## Learning points

1. CFT is less common than GIST and other gastric tumours.
2. It may be possible to diagnose CFT by considering the features found on imaging, as discussed in this paper, that is, partial calcification on CT scan and slow enhancement on dynamic contrast-enhanced studies, and extremely dark signal intensity on *T*_2_ weighted images.

## Consent

Informed consent was obtained from the patient included in this case report.
